# Physical disease in schizophrenia: a population-based analysis in Spain

**DOI:** 10.1186/1471-2458-10-745

**Published:** 2010-12-02

**Authors:** Carmen Bouza, Teresa López-Cuadrado, José María Amate

**Affiliations:** 1 Health-Care Technology Assessment Agency, Instituto de Salud Carlos III, Madrid, Spain; 2 National Epidemiology Centre, Instituto de Salud Carlos III, Madrid, Spain

## Abstract

**Background:**

Physical disease remains a challenge in patients with schizophrenia. Our objective was to determine the epidemiological characteristics and burden of physical disease in hospitalized patients with schizophrenia.

**Methods:**

We analyzed the 2004 Spanish National Hospital Discharge Registry, identified records coded for schizophrenia (295.xx) and characterized the physical diseases using the ICD-9 system and the Charlson Index. We also calculated standardized mortality ratios (SMRs) versus the general population adjusted by age and calendar time.

**Results:**

A total of 16, 776 cases (mean age: 43 years, 65% males) were considered for analysis. Overall, 61% of cases had at least one ICD-9 physical code and 32% had more than one ICD-9 code. The Charlson index indicated that 20% of cases had a physical disease of known clinical impact and prognostic significance. Physical disease appeared early in life (50% of cases were 15-31 years of age) and increased rapidly in incidence with age. Thus, for patients aged 53 years or more, 84% had at least one physical ICD-9 code. Apart from substance abuse and addiction, the most prevalent diseases were endocrine (16%), circulatory (15%), respiratory (15%), injury-poisoning (11%), and digestive (10%). There were gender-related differences in disease burden and type of disease. In-hospital mortality significantly correlated with age, the Charlson Index and several ICD-9 groups of physical disease. Physical disease was associated with an overall 3.6-fold increase in SMRs compared with the general population.

**Conclusions:**

This study provides the first nationally representative estimate of the prevalence and characteristics of physical disease in hospitalized patients with schizophrenia in Spain. Our results indicate that schizophrenia is associated with a substantial burden of physical comorbidities; that these comorbidities appear early in life; and that they have a substantial impact on mortality. This information raises concerns about the consequences and causes of physical disorders in patients with schizophrenia. Additionally, it will help to guide the design and implementation of preventive and therapeutic programs from the viewpoint of clinical care and in terms of health-care service planning.

## Background

There is growing interest in the effects of physical disease on patients with schizophrenia. Physical disease can affect psychiatric signs and symptoms, response to psychoactive drugs, life expectancy, and use of healthcare services [1-5]. However, there is no consensus about how to treat or prevent physical disease in patients with schizophrenia [6,7]. This is mainly because of the difficulties involved in selecting and analyzing representative samples of such patients [8].

In Spain, following psychiatric reform and the de-institutionalization process in the 1980 s, most people with schizophrenia live in the community and receive public universal healthcare in the same centers used by the general population [9-11]. In this scenario, our hypothesis is that the study of physical disease in hospitalized people with schizophrenia may provide relevant information for clinical practice and healthcare planning.

The objective of the present study was to examine physical disease in hospitalized people with schizophrenia, describe the epidemiological characteristics, and identify the most prevalent physical diseases as well as their impact on mortality by analysis of a national administrative database.

## Methods

This study used the National Hospital Discharge Registry of Spain, the official database of the Ministry of Health [12]. This information is derived from discharge reports from all acute-care hospitals, and is representative of the national population, as it includes data on over 90% of all annual hospital admissions nationwide.

The registry, mandated by law, includes demographic data; clinical data, including diagnoses (one main or primary diagnosis and up to 12 additional diagnoses all of which are considered as secondary diagnoses) coded according to the International Classification of Diseases, 9^th ^Revision, Clinical Modification (ICD-9-CM); dates of admission and discharge; type of admission; and characteristics and disposition upon hospital discharge.

We used population figures from the 2003-04 National Health Survey [13] to compare physical-disease prevalence between our study population and the general population. We also obtained demographic national population data from the National Statistics Institute [13].

### Period of analysis

We used data from the national database that covered the period January 1 to December 31, 2004.

### Case selection

Cases were selected by identification of codes corresponding to schizophrenia (ICD-9 codes 295.xx), among hospitalized subjects aged ≥15 years. Afterward, to avoid an overestimation of comorbidities and outcomes for each case, we performed a process of filtering and depuration of the database in which we explored the number of hospital discharges in the analysis period. To carry this out, the following identification variables were chosen: birth date, gender, admission date, discharge date, postal code and readmission (coded as a binary variable). In the database refinement process used in this study, 1,776 cases were identified, within the analysis period, as having been readmitted into the same hospital and with the same main diagnosis. Subsequently, among the admissions identified for each patient, the most complete one, in terms of coding with respect to the coding for the main diagnosis and the secondary ones, was chosen.

### Comorbidities

For each case, physical disease was characterized by ICD-9 codes: infectious diseases (001-139); neoplasms (140-239); endocrine diseases (240-279); hematological diseases (280-289); neurological diseases (320-389); diseases of the circulatory system (390-459); respiratory diseases (460-519); diseases of the digestive system (520-579); diseases of the genitourinary tract (580-629); complications of pregnancy, childbirth, and the puerperium (630-677); diseases of the skin and subcutaneous tissue (680-709); diseases of the musculoskeletal system and connective tissue (713-739); and injury and poisoning (800-999). Within each category of physical illnesses, those considered of special clinical relevance, such as Chronic Obstructive Pulmonary Disease (COPD); Ischemic Heart Disease (IHD); Myocardial Infarction (MI) and Diabetes will be analyzed specifically.

In addition, specific codes were used to identify abuse or dependency on drugs (codes ICD-9: 304.8, 304.2, 305.9), alcohol (305.0, 303.9), and tobacco (305.1, 989.84, E869.4) given their known capacity for generating or complicating the course of physical disease in patients with schizophrenia [14]. Finally, to determine the extent of physical comorbidities of known prognostic value, we used a validated ICD-9 version [15] of the Charlson comorbidity index [16]. In accordance with prior literature [17], four different score groups (0, 1-2, 3-4, > 4) were employed.

### Ethical issues

The study was exempt from institutional review board approval, because only de-identified administrative data were used.

### Data analysis

This descriptive study analyzed the prevalence (and 95% CIs) of selected physical morbidities and their distribution according to gender and age quartiles. Data are summarized as frequencies and percentages for categorical variables. Continuous variables are presented as means and standard deviations (SD). For between-group comparisons, we used χ^2 ^tests for categorical data. Continuous variables were analyzed with Snedecor's F test or the Mann-Whitney U test. Odds ratios (ORs) with 95% confidence intervals were computed where appropriate. We used an exploratory logistic regression analysis to identify the impact of physical illness upon in-hospital mortality.

The age-and gender-standardized rates were calculated by direct standardization based on the 2004 Spanish population aged ≥15 years [18].

We estimated expected values for mortality by 10 years-age groups based on the 2004 Spanish population aged ≥15 years. Observed and expected numbers of deaths were used to calculate the standardized mortality ratios (SMRs) versus the general population [2]. In order to explore the impact of physical disease on the risk of death, SMRs were calculated for the subgroups of cases with/without ICD-9 codes of physical disease.

All analyses were performed with SPSS version15.0 for Windows (SPSS Inc., Chicago, IL). A p-value < 0.05 was considered significant.

## Results

Of the 3,951,214 hospital discharges registered in subjects ≥15 years of age across the nation in 2004, and after carrying out several depuration processes for the database, 16776 records with schizophrenia were eligible for analysis (incidence rate 46.23 cases per 100,000 population/year). Of the total number of cases, 64% of hospitalisations (n = 10745; 29.6 cases per 100,000 population/year) appeared to be directly associated with schizophrenia, inasmuch as schizophrenia is shown as the principal diagnosis, whereas in the remaining 36% (n = 6031; 16.63 cases per 100,000 population/year), the primary cause of hospitalisation was stated to be some other disease.

**Figure 1:**
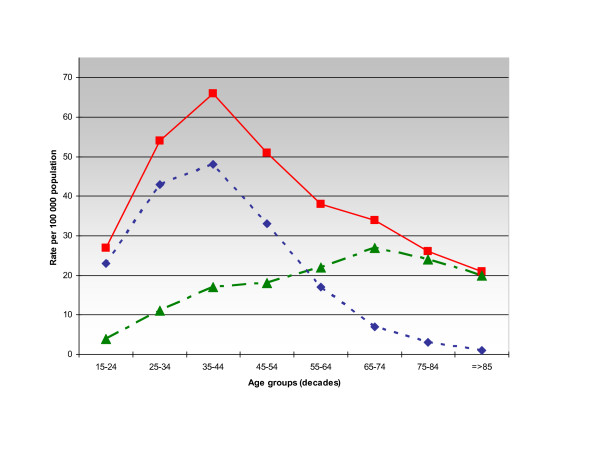
**Hospital admissions in patients with schizophrenia**. Rates of hospitalizations in patients with schizophrenia in Spain according to 10-year age groups and principal diagnosis at discharge. Overall rate (filled squares/solid line), data for cases with principal diagnosis of schizophrenia (filled diamonds/dotted line), data for cases admitted due to physical disease (filled triangles/dotted line).

Stratification by age-groups is shown in Figure 1.

As shown in Table 1 hospital admissions were mostly for acute conditions through emergency departments and more frequent in men. Mean age of the cases was 43 years and men were significantly younger than women (41.2 ± 14.7 years *vs*. 47.9 ± 17 years; p < 0.001).

**Table 1:** Global characteristics of the population

	Total	Men	Women
Cases	16776	10952 (65%)	5818 (35%)

Hospital admission:			
Acute	14 699 (88%)	9625 (88%)	5069 (87%)
Scheduled	2066 (12%)	1320 (12%)	745 (13%)

Schizophrenia in principal diagnosis at hospital discharge	10 745 (64%)	7209 (66%)	3532 (61%)

Age, years	43.5±16	41.2±14.8	47.9±17.2

Age, decades			
15-24 years	1483 (8.8%)	1149 (10.5%)	333 (5.7%)
25-34 years	4054 (24,2%)	2938 (26.8%)	1114 (19.1%)
35-44 years	4560 (27.2%)	3122 (28.5%)	1437 (14.7%)
45-54 years	2784 (16.6%)	1754 (16%)	1030 (17.7%)
55-64 years	1699 (10.1%)	963 (8.8%)	735 (12.6%)
65-74 years	1350 (8%)	718 (6.6%)	632 (10.9%)
75-84 years	680 (4.1%)	270 (2.5%)	409 (7%)
≥85 years	166 (0.99%)	38 (0.3%)	128 (2.2%)

Mean ICD-9-CM codes	1.22±1.38	1.20±1.34	1.26±1.46

Number of codes:			
1	4914 (29%)	3482 (31.8%)	1430 (24.6%)
2	2549 (15%)	1660 (15.2%)	889 (15.3%)
3	1451 (9%)	879 (8.0%)	571 (9.8%)
4	771 (5%)	461(4.2%)	310 (5.3%)
≥5	548 (3.3%)	330 (3.1%)	218 (3.8%)

Mean Charlson Index	0.43±1.26	0.43±1.28	0.43±1.20

Charlson Index (categorized)			
0 points	13 518 (81%)	8912 (81.4%)	4602 (79.1%)
1,2 points	2559 (15%)	1561 (14.3%)	996 (17.1%)
3,4 points	274 (2%)	183 (1.7%)	91 (1.6%)
>4 points	425 (2.5%)	296 (2.7%)	129 (2.2%)

Data are shown as mean ± standard deviation or number of cases and percentages (%)

The differences between the some of the gender data and the total population are due to missing values.

The mean number of physical ICD-9 codes was 1.22 ± 1.38 (range 0-8), and women had significantly more codes than men (1.26 ± 1.4 *vs*. 1.20 ± 1.3; p = 0.002). Overall, 61% of patients had at least one ICD-9 code, and 32% had more than one ICD-9 code. Stratification by age showed that 50% of cases were younger subjects (15-31 years of age), and that the number of ICD-9 codes increased with age. Thus, for patients aged 53 years or more, 84% had at least one physical ICD-9 code (17% had one code; 24% two codes; and 43% three or more codes). This increase with age occurred for both men and women.

Furthermore, 20% of the cases had Charlson indices greater than zero, although there was no statistically significant difference between the genders. The severity of this index rose significantly with age, independent of gender.

Addiction to drugs, alcohol, or tobacco was the most significant problem, and about one-third of cases had a code indicating substance abuse or dependency (Table 2). With respect to defined ICD-9 groups, the most frequent were endocrine problems, circulatory and respiratory diseases, and injury-poisoning. Within these categories, diabetes mellitus and chronic obstructive pulmonary disease (COPD) were pre-eminent, with an overall prevalence of 8% and 5.5%, respectively. Ischemic heart disease was present in 338 cases: of these, 185 had myocardial infarction, corresponding to an overall prevalence of 1.1% (95% CI: 0.94-1.26).

**Table 2:** Frequency of specific ICD-9 physical diseases

ICD-9-CM groups	No. cases, % and (95% CI)
Substance abuse/dependency	4899, 29 (28.5-29.9)
Tobacco	2533, 15 (13.6-16.4)
Alcohol	1745, 10.4 (9.9-10.9)
Drugs	2377, 14.2 (13.6-14.7)

Endocrine, nutritional and metabolic diseases	2772, 16.5% (15.9-17.1)
Diabetes	1339, 8% (6.54-9.44)

Diseases of the Circulatory System	2458, 14.7% (14-15.2)
Ischemic heart disease	338, 2% (0.55-3.6)
Cardiac failure	260, 1.5% (1.36-1.74)
Cerebrovascular Disease	257, 1.5% (1.35-1.72)

Diseases of the Respiratory System	2056, 12.3% (11.8-12.8)
COPD	926, 5.5% (5.17-5.87)

Injury-Poisoning	1901, 11.3% (9.89-12.73)

Diseases of the Digestive System	1749, 10.4% (9.96-10.9)
Hepatic	57, 0.3% (0.25-0.43)

Diseases of the Nervous System	1401, 8.4% (7.9-8.8)
Dementia	130, 0.8% (0.64-0.91)

Diseases of the Genitourinary System	1168, 7% (6.6-7.4)
Renal Insufficiency	172, 1% (0.87-1.18)

Infectious and parasitic diseases	1095, 6.5% (6.2-6.9)
AIDS	190, 1.1% (0.97-1.29)

Neoplasms	746, 4.4% (4.1-4.8)

Diseases of the Blood and blood-forming organs	724, 4.3% (4.0-4.3)

Diseases of the Skin and Subcutaneous Tissue	540, 3.2% (2.9-3.5)

Diseases of the Musculoskeletal & Connective Tissue	552, 3.3% (3.0-3.6)

Complications of pregnancy, childbirth and the puerperium	113, 1.94% (1.6-2.3)

ICD-groups are ordered by descending counts. CI95%: Confidence interval

The number of cases in all ICD-9 groups increased with age. In the case of endocrine and circulatory diseases, almost 40% of the population over the age of 53 was affected. In addition, there were gender-related differences in the prevalence of several ICD-9 groups (Table 3).

**Table 3:** Frequency of physical diseases in males and females.

ICD-9-CM groups	Men (% of cases)	Women (% of cases)	**OR**^1 ^**(95% CI), p-value**
Substance abuse/dependency	37	15	3.28 (3.02-3.56), <0.001
Tobacco	13.8	3.9	3.9 (3.4-4.5), <0.001
Alcohol	19	5.1	4.35 (3.9-4.9), <0.001
Drugs	18	9.9	1.9 (1.8-2.2), <0.001

Endocrine, nutritional and metabolic diseases	13.6	22.1	0.55 (0.51-0.6), <0.001
Diabetes	6.3	11	0.57 (0.51-0.64), <0.001

Diseases of the Circulatory System	13.2	17.4	0.7 (0.66-0.8), <0.001
Ischemic heart disease	2.3	1.4	1.67 (1.3-2.15), <0.001
Cardiac failure	1.3	1.9	0.74 (0.58-0.96), 0, 019
Cerebrovascular Disease	1.2	2.2	0.54 (0.42-0.7), <0.001

Diseases of the Respiratory System	12.7	11.5	1.12 (1-1.2), 0.022
COPD	6.4	3.8	1.74 (1.5-2.03), <0.001

Injury-Poisoning	10.6	12.7	0.82 (0.74-0.91), <0.002

Diseases of the Digestive System	10.7	9.9	1.09 (1-1.2), 0.106
Hepatic	0.9	0.3	2.7 (1.65-4.42), <0.001

Diseases of the Nervous System	7.8	9.4	0.8 (0.73-0.91), <0.001
Dementia	0.5	1.3	0.36 (0.26-0.52), <0.001

Diseases of the Genitourinary System	5.6	9.5	0.57 (0.5-0.64), <0.001
Renal Insufficiency	1	1.1	0.87 (0.64-1.2), 0.39

Infectious and parasitic diseases	6.9	6.9	1 (0.88-1.14), 0.99
AIDS	1.3	0.8	1.7 (1.23-2.4), <0.001

Neoplasms	3.8	5.7	0.65 (0.56-0.75), <0.001
Diseases of the Blood and blood-forming organs	3.5	5.9	0.57 (0.49-0.66), <0.001

Diseases of the Skin and Subcutaneous Tissue	3.1	3.4	0.9 (0.75-1.08), 0.245

Diseases of the Musculoskeletal and Connective Tissue	2.6	4.5	0.65 (0.56-0.75), <0.001

^1^OR for men versus women. OR: Odds Ratio. CI: Confidence interval

Approximately 13% of cases (n = 2,210) underwent surgical procedures during hospitalization, where digestive (n = 349) and musculoskeletal (n = 351) procedures were the most common.

A comparison between our data and the official data for the Spanish population provided by the National Health Survey [13] indicate that diabetes rates are clearly higher, twice as high in the case of women, than those observed for the general Spanish population (5.02% overall, 5.29% in women and 4.73% in men). The frequency of neoplasms observed in our study is also higher than that reported in the population data for Spain (2.37% overall, 1.92% in men and 2.79% in women). Similarly, our cases register a higher rate of tobacco and alcohol abuse/dependence than does the general Spanish population with 12.8% and 2.55% of the population (4.5% of men and 0.6% of women) being classified as heavy smokers and excessive drinkers respectively, and also a high rate of AIDS (1.61 per 1000 population). On the other hand, we found no pronounced differences in the frequency of COPD (5.33% in the overall Spanish population) and ischemic heart disease (2.39% overall, 3.2% in men and 1.62% in women).

Regarding outcomes, 88% of the cases (n = 14,701) returned home; 6.2% (n = 1,036) were transferred to other hospitals; 2.1% (n = 356) were transferred to a socio-health care center; and 2.3% (n = 387) died in hospital. No significant differences were observed in hospital mortality rates between women and men (2.6% *vs*. 2.1%; OR: 1.22, 95% CI: 0.99-1.50). The mean age of patients who died was 63 years. For men and women there were statistically significant differences in mortality according to age, with a rise in mortality after the age of 40. The mortality in cases without an associated physical illness is 0.2% (n = 12) while that in cases with one or more codes of physical disease is 3.7% (n = 375).

Furthermore, cases with schizophrenia as the main discharge diagnosis presented a hospital mortality of 0.3% (n = 27 cases), whereas that in cases with physical illness in the primary diagnosis was of 6% (n = 360 cases). The distribution of the main discharge diagnoses by gender is shown in Table 4.

**Table 4:** In-hospital deaths: Main discharge diagnosis by gender

ICD-9-CM main diagnostic categories at discharge	Number of cases		
	**Total (n = 387)**	**Men (n = 235)**	**Women (n = 152)**

Schizophrenia	27	21	6

Physical disease:			

Diseases of the Circulatory System	73	36	37

Diseases of the Respiratory System	71	45	26

Neoplasms	68	46	22

Injury-Poisoning	43	21	22

Diseases of the Digestive System	36	22	14

Infectious and parasitic diseases	19	15	4

Diseases of the Nervous System	12	9	3

Endocrine, nutritional and metabolic diseases	11	4	7

Diseases of the Genitourinary System	7	4	3

Diseases of the Musculoskeletal and Connective Tissue	5	4	1

Diseases of the Blood and blood-forming organs	5	2	3

Others. Miscellaneous	10	6	4

To investigate factors associated with in-hospital mortality, we performed an exploratory logistic regression analysis which included gender, age-quartiles, ICD-9 main diagnostic blocks at discharge, and categorized Charlson index scores. The results indicated that the risk of in-hospital death was significantly correlated with age, Charlson index score, and several main ICD-9 codes of physical disease (Table 5). There were no significant interactions between these variables (p = 0.094). Moreover, there were no significant differences by gender in mortality after controlling for age, Charlson Index, and ICD categories.

**Table 5:** Factors related to in-hospital mortality.

	Exp (B) (95% CI Exp B), p value
**Gender**	
**Male**	Reference group
**Female**	1.01 (0.81-1.27); 0.87
	
**Age range (quartiles)**	
**15-31 years**	Reference group
**32-40 years**	1.49 (0.76-2.94); 0.246
**41-53 years**	2.67 (1.45-4.91); 0.002
**>53 years**	6.14 (3.40-11.06);<0.001
	
**ICD-9-CM main diagnostic categories at discharge**	
**Diseases of the Respiratory System**	3.54 (2.8-4.48);<0.001
**Neoplasms**	2.17 (1.52-3.11);<0.001
**Diseases of the Circulatory System**	2.16 (1.70-2.75);<0.001
**Infectious diseases**	1.80 (1.36-2.38);<0.001
**Diseases of the Digestive System**	1.77 (1.39-2.26); <0.001
**Injury and Poisoning**	1.57 (1.19-2.07); <0.001
**Diseases of the Nervous System**	1.45 (1.1-1.89); 0.007
	
**Charlson Index (categorized)**	
**0 points**	Reference group
**1-2 points**	1.33 (1.01-1.76);0.045
**3-4 points**	1.76 (1.21-2.78); 0.01
**>4 points**	3.55 (2.29-5.52);<0.001

CI: Confidence interval.

**Figure 2:**
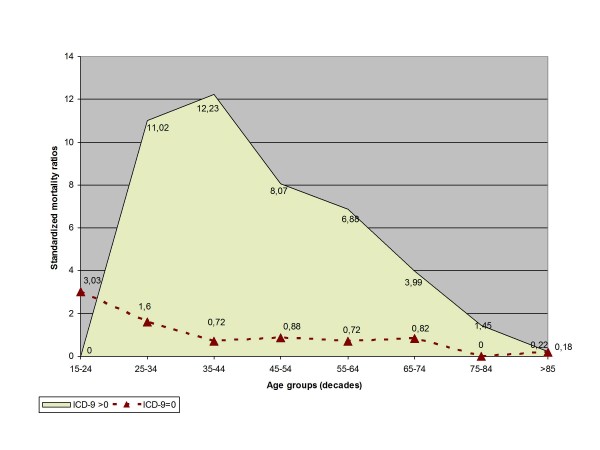
**Standardized mortality ratios**. Standardized mortality ratios (directly standardized to the 2004 Spanish national population aged ≥15 years) in hospitalized patients with schizophrenia according to 10-year age groups. Data for cases with at least one ICD-9 code of physical disease (filled area); data for cases without ICD-9 codes of physical disease (filled triangles/dotted line).

Our study found 387 observed deaths, whereas expected deaths based on general population estimates for the same calendar time amount to 167 [13]. The calculated average SMR is 2.32 (95%CI: 2.09-2.56). Furthermore, the excess over expected mortality relative to the general population was disproportionately higher in the subgroup of cases with physical disease (Figure 2). In fact, the calculated overall SMR in this group of cases is 3.68 (95%CI: 3.31-4.07).

## Discussion

This study is, to the best of our knowledge, one of the first to provide nationally representative estimates of the prevalence and characteristics of physical disease in hospitalized patients with schizophrenia in Spain. Our results indicate that schizophrenia is associated with a substantial burden of physical comorbidities; that these comorbidities appear early in life; and that they have a severe impact on mortality. Also, in agreement with prior reports [3,4,19,20], our data indicate that hospitalized schizophrenic individuals often have numerous physical diseases, and that several of these diseases are of known clinical severity and prognostic relevance, however, the study design do not allow us for making causal inferences.

As for the most prevalent ICD-9 groups, our data were in general agreement with international figures. Thus, the rates of endocrine, circulatory, respiratory, and digestive diseases in our population were comparable to those in other studies that analyzed whole populations or used administrative data [19,21,22]. Certain physical diseases (substance abuse, injury-poisoning, and infections such as AIDS) were much more frequent in young people [20,23]. In contrast, circulatory and endocrine diseases were more common in patients over the age of 40 years.

Our data also identify gender-related differences in the physical diseases of patients with schizophrenia. Thus, compared with women, men had significantly higher rates of substance abuse (alcohol, drugs, and cigarette-smoking [24]), and a higher prevalence in certain diagnostic groups, such as chronic respiratory processes, digestive diseases, and infectious diseases [6,23,25]. Although men had a lower overall risk of circulatory disease, they suffered more from ischemic heart disease and myocardial infarction. On the other hand, women were more likely to have endocrine diseases, musculoskeletal and connective tissue diseases, neurological diseases, and neoplasms, possibly related to their older age [25-27].

The results of our study add to the existing controversy about differences in the rates of physical illnesses in patients with schizophrenia compared to the general population [1,7]. Thus, a comparison of our data and official figures for the Spanish general population provided by the National Health Survey [13] suggests that subjects with schizophrenia have higher rates of substance abuse/dependency, diabetes mellitus, digestive diseases, neoplasms, and AIDS. On the other hand, we found no pronounced differences in the frequency of COPD and ischemic heart disease. These findings, though noteworthy in view of the high prevalence of related risk factors, such as diabetes and smoking, are comparable to results of previous studies. Thus, Carney and colleagues [19] found that whereas a somewhat higher percentage of persons with schizophrenia had ischemic heart disease than did controls from the general population (2.3% *vs*. 1.9%), the adjusted odds ratio was not significant. Regarding COPD, our data also agree with previous reports [25]. However, it should be noted that diagnosis of early-stage COPD can be difficult [28] and the reported rate of COPD may be biased by a failure to perform diagnostic spirometry [29]. In addition, a tendency to ignore a diagnosis of COPD in patients with schizophrenia has been reported [30].

Concerning mortality, this study highlights the impact of physical disease on the risk of death in people with schizophrenia. Our analysis indicates that the Charlson index score and the presence of certain physical diseases (*e.g.*, respiratory, circulatory, tumoral, infectious, digestive, and injury-poisoning) significantly increase the risk of death during hospitalization. In addition, our data underscore that physical disease in schizophrenia was associated to disproportionately high mortality risks relative to the general population. These results, that agree with prior reports [2,31-33], raise concerns about the consequences and causes of physical disorders in patients with schizophrenia and identify a compelling need for a specific approach aimed to detect physical comorbidities, especially those that are most common and closely related to mortality. In this regard, the high risk of mortality from respiratory diseases in our population suggests that a specific approach be used to monitor and control modifiable risk factors, such as smoking [34]. Furthermore, as the prevalence and the type of physical comorbidity show significant differences between both genders, the preventive and therapeutic measures to reduce such a disease burden and associated mortality must, in addition, have a specific gender orientation.

Our study was observational, and thus we cannot definitely identify the influence of factors linked to lifestyle, adverse drug effects, or socio-economic level to explain the pattern of physical diseases in our population. Likewise, it is impossible for us to analyze the adequacy of healthcare received by the patients. Several authors have suggested that people with schizophrenia may receive inadequate medical treatment and experience inequalities and difficulties in accessing various medical procedures, even when free and universal healthcare is available, as in Spain, at the point of care [35,36].

The results of this study extend previous work by providing a comprehensive overview of medical disorders associated with schizophrenia. In particular, our study has several strengths compared to previous reports: (a) we characterized physical diseases in a large population-based representative sample of patients with schizophrenia; and, (b) we included all diseases and objectively classified them according to the ICD-9 system. In addition, our data represent clinical practice patterns for professionals nationwide, allowing us to generalize the results. Furthermore, the use of the Charlson comorbidity index, which is widely used to predict hospitalization outcome, increases the validity of observations.

We must also acknowledge possible limitations of our work. This study is subject to the limitations inherent in retrospective studies using administrative databases. Data from these databases lack of many measures obtainable only from chart review or survey with the attendant potential for omitting important prognostic factors. Furthermore, these data do not allow causal inferences to be made. However, the use of such databases is well-established in psychiatric epidemiology and health services research and has been shown to furnish valuable information for assessing the need for preventive and therapeutic care and for service planning [37-39]. Additionally, the Spanish Ministry of Health [12] systematically performs assurance audits of the National Hospital Discharge Registry to verify coding adequacy. Moreover, we followed the guidelines for reporting observational studies, as outlined by the STROBE Initiative [40].

We also recognize that the presence of a control group would have resulted in more far-reaching results and a more precise determination of the risk and time of development of physical comorbidities in patients with schizophrenia. Currently, however, it was not possible for us to consider a sufficiently large control group representative of the patients studied. Nevertheless, given that, to our knowledge, this work constitutes the first study with this range of diagnosis and population size carried out in our country, we hope that our results will be the base of other studies with a stronger methodological design.

Finally, it is inarguable that hospitalized subjects will have a disease load and severity greater than the outpatient population. However, our results clearly show the distribution and prevalence of different physical diseases that can contribute to the deterioration of health, causing patients to be admitted into hospital and increasing their risk of death. As with several recent studies [36,41], our results have implications for the design of preventive and therapeutic programs and services for people with schizophrenia that aim to reduce the prevalence and negative impacts of physical diseases in this population.

## Conclusions

In summary, we analyzed a nationwide database to determine the prevalence and characteristics of physical diseases in hospitalized patients with schizophrenia. Our results indicate that physical illness is a major burden for such patients, that these comorbidities appear early in life and that they have a serious impact on mortality. This information raises concerns about the consequences and causes of physical disorders in patients with schizophrenia and may prove useful in the design and implementation of preventive and therapeutic programs and for health-care service planning.

## Competing interests

The authors declare that they have no competing interests.

## Authors' contributions

Authors CB and JM Amate designed the study, wrote the protocol, and managed literature searches and analysis. Authors CB and TL performed the statistical analysis. Author CB wrote the first draft of the manuscript. All authors contributed to and have approved the final manuscript.

## Pre-publication history

The pre-publication history for this paper can be accessed here:

http://www.biomedcentral.com/1471-2458/10/745/prepub
